# Psychometric evaluation of the Korean version of the Hepatitis B Quality of Life Questionnaire

**DOI:** 10.1371/journal.pone.0213001

**Published:** 2019-02-27

**Authors:** Yeonsoo Jang, Sang Hoon Ahn, Kyunghwa Lee, Jiyeon Lee, Jeong Hyun Kim

**Affiliations:** 1 College of Nursing, Mo-Im Kim Nursing Research Institute, Yonsei University, Seoul, Korea; 2 Department of Internal Medicine, College of Medicine, Yonsei University, Seoul, Korea; 3 Department of Nursing, Graduate School, Yonsei University, Seoul, Korea; McMaster University, CANADA

## Abstract

**Purpose:**

The purpose of this study was to conduct a psychometric evaluation of the Korean version of the Hepatitis B Quality of Life Questionnaire (K-HBQOL), which is designed to assess the quality of life of patients with the hepatitis B virus (HBV).

**Methods:**

The K-HBQOL was developed by converting the original English version to Korean using a back-and-forth translation method. The translated questionnaire was distributed to 168 adults with HBV. Descriptive statistics were used to summarize the demographic characteristics of these participants. Confirmatory factor analysis and exploratory principal components analysis (CFA and PCA) were performed, applying varimax rotation and using Kaiser’s eigenvalue-greater-than-one rule to examine the factor structure. The minimum average partial rule was also used to identify the number of components.

**Results:**

The original factor model was not confirmed by CFA in this sample. Six components (Psychological Well-being, Stigmatization, Anticipation Anxiety, Weakness, Vitality, and Vulnerability) were extracted by PCA, with 69.11% of the total variance explained. In this process, one component present in the original factor model (Transmission) was not found, while another component (Weakness) was extracted.

**Conclusions:**

This study revealed the psychometric characteristics of the Korean version of the HBQOL for patients with hepatitis B. We suggest a study with a larger sample is needed to evaluate validity and reliability of the K-HBQOL for other Korean populations.

## Introduction

Hepatitis B is one of the most common diseases in the world, and it is a major cause of cirrhosis, liver failure, and liver cancer. According to a 2017 report from the World Health Organization (WHO), approximately 250 million people worldwide are infected with the hepatitis B virus (HBV), while over 800,000 people die annually from complications related to the virus [1]. Over the last 10 years, the prevalence of the hepatitis B surface antigen (HBsAg) in Korea has declined significantly, mainly as a result of a nationwide vaccination project; however, over four percent of Korean adults in their 30s and over remain positive for HBV. In particular, Korean men aged from their 30s to their 50s have a higher sero-prevalence than Korean women [2]. HBV can also have several indirect effects, as the socio-economic burden of patients who are members of the working population may be increased [3, 4], and these socio-economic problems, along with the physical and psychological issues associated with HBV, might confer negative effects on sufferers’ quality of life (QOL).

Health-related quality of life (HRQOL) is a construct that measures patients’ self-perceptions concerning their physical, psychological, and social health [5]. It is important to monitor the self-reported HRQOL of patients with chronic HBV because this concept facilitates not only the measurement of the various aspects of the patient’s burden, but also provides valuable information concerning patients’ risk factors. Therefore, healthcare providers should consider the relationship between HRQOL and health outcomes in patients with chronic disease, such as HBV, and thereby provide healthcare service that can improve health outcomes [3].

Spiegal and his colleagues developed and validated the Hepatitis B Quality of Life Questionnaire (HBQOL) in order to measure the HRQOL of patients with HBV [6]. Before the development of the HBQOL, research into the QOL of patients with hepatitis required applying general QOL instruments. However, this method was limited; although the general QOL instruments are suitable for comparing the QOL of patients with HBV with that of other populations, it is not completely suitable for measuring the disease-related characteristics of QOL in patients with HBV. Consequently, the HBQOL was developed to assess specific and crucial aspects of HRQOL in patients with HBV [6].

HRQOL is influenced by cultural background. Although culture is not an attribute or dimension of QOL, QOL influences the decision-making or health behaviors of individuals, especially patients with chronic diseases [7]; therefore, HRQOL measurements must be comprehended within social and cultural contexts [8, 9]. After development and validation of the original instrument, versions of the HBQOL in other languages were validated [10]; however, there is no extant psychometric evaluation in Korean populations. To understand the QOL of Korean patients with HBV, use of the HBQOL is necessary; thus, the instrument should be validated among Koreans with HBV. Therefore, this study conducted psychometric evaluations of the K-HBQOL.

## Methods

### Design and participants

We performed data collection between March 2015 and May 2015 at the outpatient liver clinic of a university hospital located in Seoul, Korea. When selecting participants, inclusion criteria were: 1) aged 18 years or older, 2) having been diagnosed with HBV more than three months after detection of HBsAg, more than six months previously, 3) having been free of liver cirrhosis, hepatocellular carcinoma, other infections (e.g., gastritis, Crohn’s disease), or immune diseases (e.g., systemic lupus erythematosus, human immunodeficiency virus) for at least one month, and 4) able to give informed consent.

The required sample size was determined by choosing to have a ratio of five individuals per item [11]. Applying this criterion to our study, it was determined that a minimum of 155 participants was required for the factor analysis, as the HBQOL contains 31 items. Convenience sampling was then applied at the study site; a total of 168 individuals participated.

### Study instruments

#### Hepatitis B Quality of Life (HBQOL)

The original HBQOL consists of 31 items divided into six factors: Psychological Well-being (8 items), Anticipation Anxiety (6 items), Vitality (5 items), Stigmatization (6 items), Vulnerability (3 items), and Transmission (3 items). Each item is ranked using a five-point Likert scale (from 1–5); the total score is then converted into between 0 and 100 points, using scoring instructions provided by the original author. A high score represents high QOL. Cronbach’s α in the original study was 0.98 [6].

#### Translation process

The Korean version of the HBQOL was generated by translation from English to Korean using the back-and-forth method [12, 13], once translation approval had been obtained from the authors of the original instrument. To perform translation, two bilingual master’s level nursing researchers, who each had over three years of clinical experience, initially translated the instrument from English into Korean. After translation from English to Korean, a bilingual master’s level nurse performed a back-translation into English. Then, the back-translated version was compared with the original version by a native English speaker, to evaluate the differences between the two versions. After the comparison, we conducted a preliminary survey of six patients. Through this preliminary survey, we were able to determine whether the content was clear, whether there were any terms that were difficult to understand, and how long it took to respond to the questionnaire. Finally, a medical doctor and a nurse, each of whom had over five years of experience in the division of hepatology, confirmed the applicability of the final Korean version of the HBQOL.

### Data analysis

Data were analyzed using SPSS 23.0 (Chicago, IL, USA) and AMOS 23.0 (Chicago: IBM SPSS). Descriptive statistics were used to characterize the demographics of the participants. The distributions of the scores of all items of the HBQOL were summarized by means and standard deviations. Floor and ceiling effects were assessed by examining the skewness of the data; if 15% or more of the participants were allocated to either extreme, the data were considered to be skewed [14].

#### Confirmatory factor analysis (CFA)

CFA was carried out to determine the goodness-of-fit of our sample data to the original model [6], using the following criteria [15–18]: 1) χ^2^/degrees of freedom (DF) between 2.0 and 5.0; 2) goodness-of-fit index (GFI) and adjusted GFI (AGFI) of at least 0.9; 3) standardized root mean-squared residual (RMR) less than 0.08; 4) root mean-squared error of approximation (RMSEA) less than 0.07; 4) normed-fit index (NFI) greater than 0.95; 5) Tucker-Lewis index (TLI) greater than 0.95; 6) comparative fit index (CFI) greater than 0.95.

#### Exploratory principal components analysis (PCA)

According to previous studies, if the observed original factor structure of an instrument does not fit a new sample well when the instrument is translated and used in another language, PCA may be used to improve the model [19, 20]. An exploratory principal components analysis was performed, using the principal components method with varimax rotation. Kaiser’s eigenvalue-greater-than-one rule (K1) was used to determine the number of components to retain; subsequently, items with component loadings greater than 0.4 were retained [21]. Each component comprised at least three items. The minimum average partial (MAP) rule was used to confirm the number of components to retain.

### Ethical considerations

This study was approved by the Yonsei University Health System, Severance Hospital, Institutional Review Board (IRB #4-2014-0940), and written consent was obtained from all participants.

## Results

### Subject characteristics

A total of 168 patients participated in this study; Table 1 shows the demographic and clinical characteristics of the participants. The overall mean age was 48.59 ± 9.78 years (range: 22–69 years), and 67.9% of the patients were men. Of the 168 patients, 66.8% were college graduates, and 60.7% were employed. The mean length of diagnosis was 18.28 ± 9.90 years, and 97.6% of the patients had used anti-viral medications.

**Table 1:** Demographic and clinical characteristics (N = 168).

		Mean (SD)	n (%)
***Demographic***			
Sex	Men		114 (67.9)
	Women		54 (32.1)
Age		48.59 (9.78)	
	<40		34 (20.2)
	41–50		60 (35.7)
	51–60		53 (31.5)
	>60		21 (12.5)
Education^a^	≤ High school		55 (33.1)
	> High school		111 (66.8)
Occupation^a^	Employed		102 (60.7)
	Unemployed		62 (36.9)
Spouse^a^	Yes		140 (84.8)
	No		25 (15.1)
Monthly household income^a^ (10,000 KRW)	≤ 300 > 300		40 (24.4) 124 (75.6)
***Clinical***			
Length of diagnosis (years)^a^		18.28 (9.90)	
Antiviral therapy	Yes		164 (97.6)
	No		4 (2.4)
ALT		26.1(14.3)	
AST		28.3(23.0)	
HBV DNA	< 2000 units		160 (95.2)
	≥ 2000 units		8 (4.8)
Comorbidity^b^	Hypertension		19 (36.5)
	Diabetes mellitus		11 (21.2)
	Hyperlipidemia		8 (15.4)
	Arteriosclerosis		0 (0)
	Others		14 (26.9)

^a^ Differs from the total number of participants due to missing data

^b^ multiple replies.

### Psychometric evaluation

We tested a six-factor model based on the original structure of the HBQOL using CFA. The goodness-of-fit statistics are presented in Table 2. Among the eight criteria, seven indicated poor fit of the model to our data. That is, the original model was not confirmed in our data through CFA. Therefore, we carried out PCA to determine a structure that was more appropriate for our data.

**Table 2:** Goodness-of fit indices for the HBQOL factor model for the current data.

Index	Value
χ^2^/DF	2.97
GFI	.67
AGFI	.60
RMR	.68
RMSEA	.74
NFI	.76
TLI	.12
CFI	.11

The Kaiser-Meyer-Olkin (KMO) measure and Bartlett’s test showed that our data were suitable for PCA (KMO = .915, p < .001). Six components were extracted by PCA, which accounted for 69.11% of the total variance. Items with cross-loadings, defined as loadings that differed by less than 0.2 on two or more components [22] were F5, F8, F9, and C15. As this was first use of the HBVQOL in Korean, we named each component without eliminating items. Based on PCA results, the names of each component were as follows: component 1, “Psychological well-being”; component 2, “Anticipation anxiety”; component 3, “Stigmatization”; component 4, “Weakness”; component 5, “Vitality”; and component 6, “Vulnerability”.

Table 3 presents component loadings of each item. In comparison to the original version, 10 items moved to different components in this study: F1, F2, F10, F11, F12, C4, C6, C8, C9, and C10. In this process, one component in the original version was not replicated and a new component was extracted. That is, three items (F11, C4, C7) from the original component “Transmission” were reorganized. Along with disaggregation of the component “Transmission,” the component “Weakness” was newly derived, consisting of F10 (from the original component “Psychological well-being”), F11 (from the original component “Psychological well-being”), and F12 (from the original component “Vitality”). In comparison with the original version, our results extracted a new component and moved items to new components. Therefore, so we applied an alternative approach to re-confirm the number of components, using the MAP test. The smallest average partial value was 0.019. This test supported the presence of six components. The descriptive statistics of our data are shown in Table 4. The overall HBQOL score was 64.41 ± 18.30 in this study. Some items were skewed in our data.

**Table 3:** Components loadings for K-HBQOL (N = 168).

No.	Item Name	Component 1	Component 2	Component 3	Component 4	Component 5	Component 6
**Psychological well-bing**							
F2	Stigmatized	**.80**	.16	.32	-.09	.20	.10
F1	Ashamed	**.78**	.14	.29	-.17	.20	.11
F3	Sad	**.75**	.23	.20	.20	.11	.21
F4	Frustrated	**.72**	.21	.34	.24	.06	.08
F7	Angry	**.66**	.15	.26	.39	.01	.06
F6	Anxious	**.65**	.35	-.02	.38	.10	.15
F13	Scared	**.63**	.18	.02	.28	.10	.17
F8	Isolated from others	**.61**	.04	.49	.25	.10	.04
F9	something bad might happen	**.53**	.33	.14	.49	.09	.07
**Anticipation anxiety**							
C2	Concern: liver cancer	.18	**.85**	.20	.15	.09	.05
C6	Concern: easier to get ill	.16	**.81**	.20	.19	.13	.07
C5	Concern: flare	.21	**.80**	.18	.06	.09	.30
C1	Concern: liver failure	.30	**.78**	.20	.12	.09	.09
C15	Concern: health worsen	.23	**.54**	.14	.07	.10	.52
C4	Concern: transmit to child	.14	**.48**	.36	-.07	.15	.27
**Stigmatization**							
C11	Concern: socially isolated	.37	.11	**-.78**	.17	-.02	.16
C10	Concern: overly self-conscious	.34	.21	**-.75**	.08	.02	.23
C12	Concern: something serious wrong	.16	.21	**-.73**	.34	.04	.29
C3	Concern: someone influential	.26	.24	**-.66**	-.23	.09	-.14
C14	Concern: embarrassed	.18	.19	**-.66**	.21	.05	.41
C7	Concern: transmit to partner	.03	.37	**-.58**	.17	.21	.02
**Weakness**							
F12	Less productive	.16	.09	.22	**.73**	.34	.10
F10	Life less enjoyable	.45	.13	.12	**.65**	.11	.24
F11	Sexual activity difficult	.20	.10	.35	**.46**	.35	.10
**Vitality**							
P2	Memory problems	.08	.10	.18	-.01	**.81**	.12
P3	Muscle aches	.12	-.01	.03	.15	**.74**	.14
P1	Tiredness	.12	.28	-.01	.23	**.69**	-.02
F5	Worn out and tired	.32	.31	-.09	.43	**.49**	.08
**Vulnerability**							
C13	Concern: watch what eat	.18	.07	.13	.16	.09	**.75**
C8	Concern: watch medicines	.12	.43	.18	-.00	.18	**.61**
C9	Concern: life expectancy	.19	.28	.33	.34	.14	**.48**
	Eigenvalue	5.33	4.39	4.25	2.73	2.50	2.22
	% of variance explained	17.21	14.16	13.72	8.81	8.05	7.17
	Cumulative variance	17.21	31.36	45.08	53.89	61.94	69.11

NOTE. Abbreviations: F, frequency item; C, concern item; P. physical symptom item. Bold values indicate items with the highest loadings onto each component.

**Table 4:** Mean scores and floor and ceiling effects for each item (N = 168).

NO	Item	Mean(SD)	Floor (%)	Ceiling (%)
**Component 1 (Psychological Well-being)**				
F8	Isolated from others	85.86(22.67)	1.2	66.1
F4	Frustrated	76.19(27.88)	4.2	45.8
F2	Stigmatized	75.30(29.72)	6.5	45.8
F13	Scared	75.15(23.93	4.2	29.2
F7	Angry	74.11(29.71)	5.4	44.0
F9	something bad might happen	73.66(25.70)	3.0	34.5
F3	Sad	66.67(29.55)	6.0	30.4
F6	Anxious	65.92(28.62)	6.0	25.0
F1	Ashamed	65.03(31.33)	10.7	27.4
**Component 2 (Anticipation Anxiety)**				
C1	Concern: liver failure	48.07(28.69)	11.9	8.9
C4	Concern: transmit to child	46.28(38.66)	29.2	22.6
C6	Concern: easier to get ill	46.13(31.67)	17.3	12.5
C15	Concern: health worsen	44.20(26.94)	15.5	6.5
C2	Concern: liver cancer	43.01(31.42)	20.2	9.5
C5	Concern: flare	36.90(29.59)	24.4	6.5
**Component 3 (Stigmatization)**				
C11	Concern: socially isolated	75.45(28.23)	3.6	45.2
C12	Concern: something serious wrong	72.32(30.47)	6.0	42.3
C10	Concern: overly self-conscious	69.79(30.08)	5.4	36.9
C3	Concern: someone influential	69.64(33.63)	8.9	44.6
C14	Concern: embarrassed	68.15(30.55)	4.8	35.7
C7	Concern: transmit to partner	61.46(36.07)	13.7	35.7
**Component 4 (Weakness)**				
F11	Sexual activity difficult	84.97(23.87)	1.2	64.9
F12	Less productive	80.80(25.37)	1.8	52.4
F10	Life less enjoyable	75.60(26.66)	2.4	41.1
**Component 5 (Vitality)**				
P3	Muscle aches	78.57(24.21)	0.6	45.8
P2	Memory problems	75.89(25.65)	1.2	41.7
F5	Worn out and tired	69.49(26.87	4.8	26.8
P1	Tiredness	59.23(26.60)	4.8	13.1
**Component 6 (Vulnerability)**				
C9	Concern: life expectancy	51.93(32.37)	10.1	18.5
C13	Concern: watch what eat	48.51(26.20)	10.1	9.5
C8	Concern: watch medicines	32.59(31.16)	31.0	9.5
Overall score		64.41(18.30)		

## Discussion

This is the first study to conduct a psychometric evaluation of a Korean version of the HBQOL in patients with HBV. The results of CFA suggested that the original factor structure of the HBQOL was not a good fit to the current data; accordingly, a factor structure appropriate for the current data was obtained using PCA. Our findings are similar to those of previous studies that did not confirm the original factor models using CFA in validity tests of the translated instruments [19, 20]. When an instrument is translated from the original language to another language, CFA does not always confirm the appropriateness of the original factor model. For this reason, when an instrument is applied in different cultures and languages, the participants’ particular situation and cultural considerations are important [23].

Another interesting finding is that six components were extracted by PCA, and some items moved to different components in this study, as compared with the original factor structure. Although the same number of components remained, one component of the original instrument was not obtained and another component was newly extracted. Therefore, we confirmed the original number of components using another method. Various methods have been recommended by which to confirm the best number of components [24, 25]. MAP approaches have been suggested as suitable for deciding the number of components [25–27]. In the present study, the six components of the K-HBQOL were extracted by K1 and MAP approaches, and some items were rearranged in the PCA-derived solution as compared with the original factor structure.

The differences between the current findings and the original instrument development may be due to the different culture and characteristics of Koreans and Americans with HBV. More subjects reported viral responses than in the original study [6], and most subjects had experienced anti-viral treatment in the current study. Accordingly, the participants in this study might have experienced relatively mild HBV symptoms.

A previous study reported similar tendencies in Koreans with HBV as in the current study. Fatigue, a typical symptom of hepatitis, was low-to-moderate in patients with HBV. Additionally, the characteristics of fatigue were more psychological than physical [28]. Further, Korea has a national healthcare insurance system, whereby medical expenses related to HBV treatment can be reimbursed. These characteristics might affect adherence to anti-viral medications and, consequently, symptom severity. These observations suggest the likely characteristics of the HBQOL in Koreans with HBV.

A previous validation of the HBQOL for Iranians with HBV also reported reorganizing some items, but the original factors remained in the instrument structure [10]. Characteristics of different participant groups might explain the different instrument structures. The “Transmission” component remained in the Iranian sample. However, this component was excluded and a “Weakness” component was newly extracted in the Korean sample. In the Iranian study, educational level was lower than in the current study sample. In addition, 68.5% of the Iranian sample reported they did not know the possible transmission route of HBV. Although we did not assess this variable, our participants seemed less concerned regarding transmission of HBV. According to previous studies, perinatal transmission of HBV has decreased after implementation of a health policy program related to vaccination in Koreans [29, 30]. It has been reported that the knowledge of Koreans with respect to HBV is moderate to high [31], indicating that Koreans possess reasonable knowledge regarding the transmission of HBV. The participants of the current study might have perceived phenomena in their lives such as “less productive,” “life less enjoyable,” and “sexual activity difficult” as weakness. Further studies need to be conducted to explain the attributes of this component in Koreans with HBV.

This study has some limitations. First, a convenience sampling method was used and our data were skewed, as evidenced by floor and ceiling effects. Skewed data are commonly found when assessing quality of life [32], and our participants showed a similar pattern. However, the results must be interpreted carefully, and their generalizability is limited. Another study limitation is that a cross-sectional design was used, and reliability was not tested. The results of this study need to be confirmed through further study of large samples of Koreans with HBV. Additionally, studies are needed to examine the validity and reliability of the K-HBQOL in different populations. Therefore, we suggest the need for a larger sample study to evaluate validity and reliability of the K-HBQOL for other Korean samples.

Despite these limitations, our study is valuable in that it indicates linguistic and cultural consideration is essential when an instrument is translated and used in other languages and cultures.

## Conclusions

This study revealed the psychometric characteristics of the Korean version of the HBQOL for patients with hepatitis B. The study findings demonstrate that psychometric properties of the K-HBQOL need additional validation. We suggest that further, larger validation research studies should be conducted among diverse Korean populations with HBV in order to confirm and expand upon the current findings.
